# Effects of Nocturnal Light on (Clock) Gene Expression in Peripheral Organs: A Role for the Autonomic Innervation of the Liver

**DOI:** 10.1371/journal.pone.0005650

**Published:** 2009-05-21

**Authors:** Cathy Cailotto, Jun Lei, Jan van der Vliet, Caroline van Heijningen, Corbert G. van Eden, Andries Kalsbeek, Paul Pévet, Ruud M. Buijs

**Affiliations:** 1 Netherlands Institute for Neuroscience, Amsterdam, The Netherlands; 2 Division of Gastroenterology and Hepatology, Academic Medical Center, Amsterdam, The Netherlands; 3 Institut des Neurosciences Cellulaires et Intégratives UMR/LC2 7168, Département de Neurobiologie des Rythmes, IFR-Neurosciences, Strasbourg, France; 4 Instituto de Investigaciones Biomedicas UNAM, Ciudad Universitaria, Distrito Federal, México; 5 Tongji Medical College of Huazhong University of Science & Technology, Wuhan, People's Republic China; 6 Department of Endocrinology and Metabolism, Academic Medical Center, Amsterdam, The Netherlands; Pennsylvania State University, United States of America

## Abstract

**Background:**

The biological clock, located in the hypothalamic suprachiasmatic nucleus (SCN), controls the daily rhythms in physiology and behavior. Early studies demonstrated that light exposure not only affects the phase of the SCN but also the functional activity of peripheral organs. More recently it was shown that the same light stimulus induces immediate changes in clock gene expression in the pineal and adrenal, suggesting a role of peripheral clocks in the organ-specific output. In the present study, we further investigated the immediate effect of nocturnal light exposure on clock genes and metabolism-related genes in different organs of the rat. In addition, we investigated the role of the autonomic nervous system as a possible output pathway of the SCN to modify the activity of the liver after light exposure.

**Methodology and Principal Findings:**

First, we demonstrated that light, applied at different circadian times, affects clock gene expression in a different manner, depending on the time of day and the organ. However, the changes in clock gene expression did not correlate in a consistent manner with those of the output genes (i.e., genes involved in the functional output of an organ). Then, by selectively removing the autonomic innervation to the liver, we demonstrated that light affects liver gene expression not only via the hormonal pathway but also via the autonomic input.

**Conclusion:**

Nocturnal light immediately affects peripheral clock gene expression but without a clear correlation with organ-specific output genes, raising the question whether the peripheral clock plays a “decisive” role in the immediate (functional) response of an organ to nocturnal light exposure. Interestingly, the autonomic innervation of the liver is essential to transmit the light information from the SCN, indicating that the autonomic nervous system is an important gateway for the SCN to cause an immediate resetting of peripheral physiology after phase-shift inducing light exposures.

## Introduction

In the course of evolution, timekeeping mechanisms, such as the molecular clockwork, have evolved in all cells of the body. However, in mammals the master clock is located in the brain, in the hypothalamic suprachiasmatic nucleus (SCN) [1]. SCN neurons display spontaneous circadian rhythms of electrical activity *in vivo* as well as *in vitro* [2], [3]. The SCN distributes its rhythmic signal to all tissues of the body via hormones and the autonomic nervous system [4]–[7]. So far light is considered to be the most important synchronizer for the SCN, due to its influence on the neuronal activity of SCN neurons [8]. Nocturnal light exposure immediately affects corticosterone secretion and inhibits melatonin secretion, locomotor activity, body temperature and heart rate [9]–[13]. Although a functional SCN is required for the majority of light effects on the peripheral organs and behavior, also extra-SCN projections might be involved [14], [15]. Since nocturnal light exposure not only results in a change in pineal and adrenal clock gene expression, but also in changes in melatonin and corticosterone secretion [10], [13], [16], it has been suggested that these two hormones transmit the light signal to other tissues in order to adapt their function [17]–[19]. Especially the influence of the SCN on peripheral clock genes is thought to be transmitted by its control over corticosterone secretion [10], [18]. Nevertheless, autonomic connections between the SCN and almost every organ of the body have been demonstrated [20]–[26]. Indeed, the modulatory effect of light (via the SCN) on the autonomic nervous system (ANS) was already recognized in the early years of the discovery of the SCN [27]. Therefore, the ANS provides an alternative possibility for the SCN to transmit light information to peripheral oscillators, and consequently to adjust the organ-specific output according to the light changes in the environment.

In the present study, we focused on the immediate effects of light on peripheral organs by investigating 1) the changes in clock gene expression in different organs, 2) whether the light-induced changes in clock gene expression correlate with the functional changes of an organ and 3) whether the ANS is responsible for the light-induced changes of hepatic (clock) gene expression. In order to investigate the first two questions, (clock) gene expression patterns of several organs (i.e., adrenal, liver, pineal, heart and muscle tissue) were analyzed after light exposure at circadian time (CT) 14 and CT20. To examine the role of the ANS in light transmission, we focused on the expression of clock genes and output genes in the liver of intact and liver-denervated animals before and after a 1 h light exposure, at CT14.

## Results

### Effects of light exposure, at two different circadian times, on the gene expression in various organs

To characterize the time dependency of the light-induced changes in the peripheral oscillator we selected some clock genes essential for the clock machinery, i.e, *period* 1/2/3 (*Per1/2/3*), *cryptochrome*1/2 (*Cry1/2*), and a clock-output gene D-albumin-binding protein (*Dbp*). As specific-organ output genes, we chose arylalkylamine-N-acetyltransferase (*AANAT*) for the pineal gland, melanocortin receptor-2 (*MC2R*) for the adrenal and Glucose 6 phosphates (*G6pc*), glucokinase (*GCK*), glucose transporter 2 (*GLUT2*) and phosphoenolpyruvate carboxykinase (*PEPCK*) as glucose-metabolizing enzymes in the liver.

**Figure 1:**
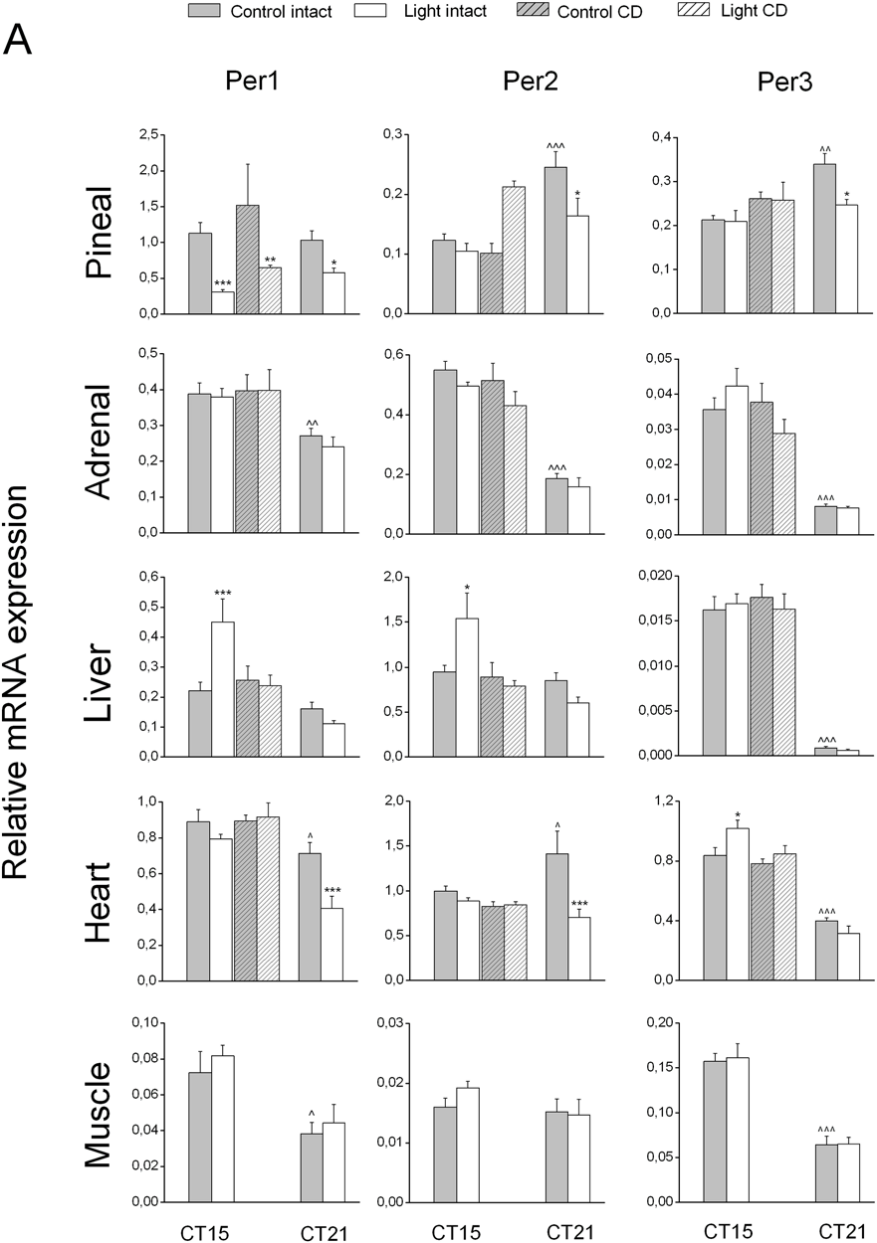
Effects of nocturnal light exposure and liver denervation on Per1, 2 and 3 gene expression. Effects of a 1 h light exposure at either CT14 or CT20 on mRNA levels of peripheral clock gene were studied in different organs of liver-intact and liver-denervated groups. Gray bars represent the non light-exposed control group (n = 8 at CT14 and n = 6 at CT20) and white bars represent the light-exposed group (n = 9 at CT14 and n = 6 at CT20). Hatched bars represent the liver denervated groups (non light-exposed group, n = 4, light- exposed group CD, n = 6). Each value is the mean±S.E.M. * P<0.05, ** P<0.01, *** P<0.005, compared to the respective non-light-exposed control group; ∧ P<0.05, ∧∧ P<0.01, ∧∧∧ P<0.005, compared to the control group at CT15.

We analyzed the expression levels of these genes by RT-PCR in 4 groups of rats, some of which were exposed to light at CT14 or CT20, and some that were kept in darkness. Animals were sacrificed at CT15 (n = 8 for controls and n = 9 for light exposed) and CT21 (n = 6 for both groups). The control group at CT15 consisted of both intact animals (n = 4) and animals that were sham-operated (n = 4). Since there were no significant differences between intact and sham-operated animals (p>0.05), both groups were analyzed as one control group. In all organs studied, the basal expression of most of the clock genes showed a clear time-dependency (**Figure 1** **&** **2** **and** **Table 1** **)**.

**Figure 2:**
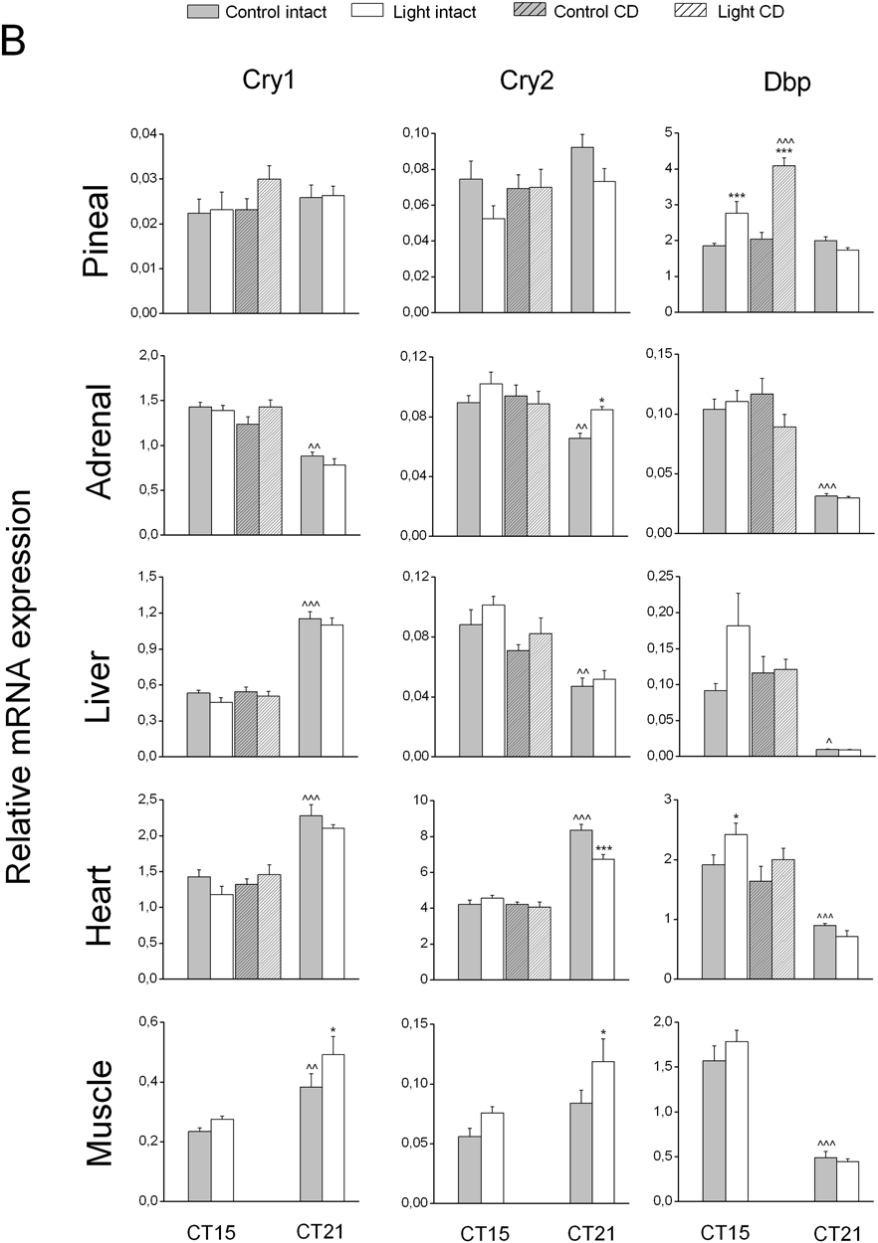
Effects of nocturnal light exposure and liver denervation on Cry1, 2 and Dbp gene expression. The effects of a 1 h light exposure at either CT14 or CT20 on mRNA levels of these clock genes were studied in the same groups mentioned in Figure 1. Each value is the mean±S.E.M. * P<0.05, ** P<0.01, *** P<0.005, compared to the respective non-light-exposed control group; ∧ P<0.05, ∧∧ P<0.01, ∧∧∧ P<0.005, compared to the control group at CT15.

**Table 1:** Statistical analysis of clock-gene expression changes comparing nocturnal light exposure at CT15 and CT21 and liver-intact *versus* liver-denervated rats with CT14–15 light exposure.

CT15 *versus* CT21							Sham *versus* Denervation						
	Per1	Per2	Per3	Cry1	Cry2	Dbp		Per1	Per2	Per3	Cry1	Cry2	Dbp
**Pineal**							**Pineal**						
Time (T)	0.407	<0.001	0.037	0.356	0.035	0.055	Dener. (D)	0.069	0.732	0.086	0.329	0.526	0.019
Light (L)	<0.001	0.016	0.051	0.854	0.026	0.143	Light (L)	<0.001	0.247	0.909	0.324	0.279	<0.001
T*L	0.095	0.109	0.069	0.964	0.874	0.016	D*L	0.907	0.930	0.996	0.436	0.255	0.080
**Adrenal**							**Adrenal**						
Time (T)	<0.001	<0.001	<0.001	<0.001	0.002	<0.001	Dener. (D)	0.729	0.153	0.653	0.297	0.565	0.737
Light (L)	0.478	0.087	0.956	0.258	0.012	0.352	Light (L)	0.928	0.055	0.359	0.285	0.651	0.883
T*L	0.673	0.554	0.829	0.617	0.572	0.282	D*L	0.912	0.677	0.261	0.111	0.261	0.195
**Liver**							**Liver**						
Time (T)	<0.001	0.009	<0.001	<0.001	<0.001	<0.001	Dener. D)	0.147	0.061	0.810	0.409	0.056	0.595
Light (L)	0.086	0.357	0.830	0.145	0.241	0.124	Light (L)	0.087	0.239	0.840	0.138	0.196	0.159
T*L	0.011	0.029	0.669	0.781	0.577	0.120	D*L	0.046	0.103	0.508	0.564	0.902	0.209
**Heart**							**Heart**						
Time (T)	<0.001	0.334	<0.001	<0.001	<0.001	<0.001	Dener. (D)	0.311	0.038	0.070	0.501	0.315	0.106
Light (L)	0.001	0.002	0.346	0.077	0.017	0.314	Light (L)	0.552	0.341	0.050	0.663	0.665	0.045
T*L	0.070	0.018	0.017	0.775	0.001	0.042	D*L	0.342	0.197	0.329	0.144	0.304	0.732
**Muscle**													
Time (T)	<0.001	0.147	<0.001	<0.001	0.002	<0.001							
Light (L)	0.409	0.466	0.853	0.031	0.014	0.513							
T*L	0.855	0.310	0.894	0.317	0.473	0.322							

Data were analyzed by a mixed one-way ANOVA: (Time (2 levels) and Light (2 levels), or (Denervation (2 levels) and Light (2 levels). If significant effects were detected, it was followed by a post-hoc LSD test. The 2 levels of Time, Light and Denervation were: CT15 *versus* CT21, light-exposure *versus* no light exposure, and control *versus* denervated, respectively.

**Figure 3:**
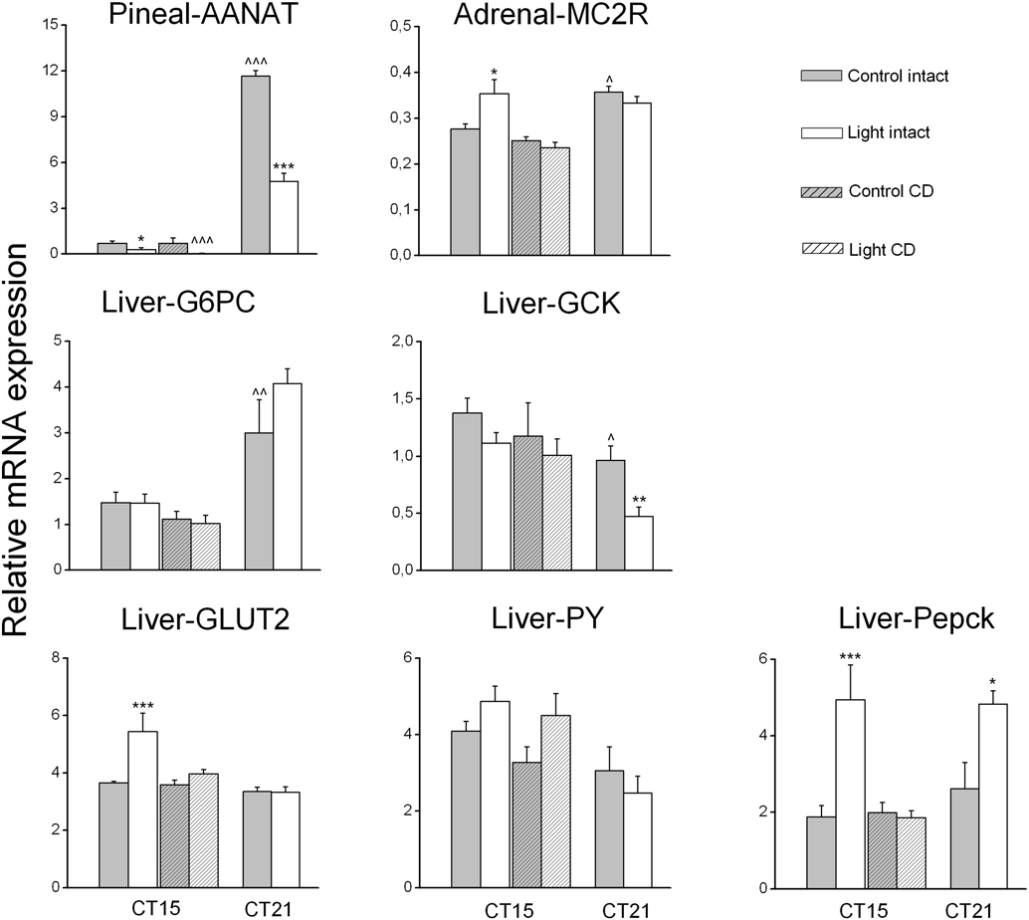
Effect of nocturnal light exposure and liver denervation on the enzyme/receptor expression of peripheral tissues. Effects of a 1 h light exposure at either CT14 or CT20 on the expression of enzymes/receptor genes in different organs of liver-intact (control) and liver-denervated (CD) animals. Gray bars represent the non-light-exposed animals (n = 8 at CT14 and n = 6 at CT20) and white bars represent the light-exposed animals (n = 9 at CT14 and n = 6 at CT20). Hatched bars represent the liver denervated groups (non light-exposed group, n = 4, light-exposed group CD, n = 6). Note that the effect of light on hepatic Per1, Per2, GLUT2 and PEPCK is absent in denervated rats. Each value is the mean±S.E.M. * P<0.05, ** P<0.01, *** P<0.005, compared to the non-light-exposed control group; ∧∧∧ P<0.005, compared to the non-denervated control group.

The diurnal changes of the peripheral clock gene expression patterns and their circadian phase observed in our study confirmed those of previous studies [16], [28]–[34]. Also some of the output genes in the liver and the pineal exhibited time-dependent changes in their expression pattern as reported previously [35], [36] (**Table 2**, **Figure 3**). Interestingly, the expression of the MC2 receptor in the adrenal also displayed a clear time-dependent change (**Figure 3**), suggesting a change in the sensitivity of the adrenal to ACTH according to the time of day, as proposed in previous studies [9], [37].

**Table 2:** Statistical analysis of output-gene expression changes comparing nocturnal light exposure at CT15 and CT21 and liver-intact *versus* liver-denervated rats with CT14–15 light exposure.

CT15 *vs* CT21	Pineal	Adrenal	Liver				
	AANAT	MC2R	G6pc	GCK	GluT2	PY	PEPCK
Time (T)	<0.001	0.189	<0.001	<0.001	0.009	<0.001	0.653
Light (L)	<0.001	0.256	0.164	0.003	0.048	0.829	<0.001
T*L	<0.001	0.035	0.155	0.331	0.044	0.133	0.544
**Sham ** ***vs*** ** Dener.**							
Dener. (D)	0.055	0.008	0.081	0.323	0.116	0.191	0.043
Light (L)	0.034	0.223	0.808	0.163	0.031	0.033	0.045
D*L	0.632	0.073	0.850	0.748	0.147	0.617	0.030

Data were analyzed by a mixed one-way ANOVA: (Time (2 levels) and Light (2 levels), or (Denervation (2 levels) and Light (2 levels). If significant effects were detected, it was followed by a post-hoc LSD test. The 2 levels of Time, Light and Denervation were: CT15 *versus* CT21, light-exposure *versus* no light exposure, and control *versus* denervated, respectively.

### Response of pineal and adrenal to light exposure

As shown in **1&2**, light exposure induced significant changes in the mRNA expression of clock genes in the adrenal and pineal as previously described [10], [13], [16]. In the pineal, Per1 expression decreased during both CT14 and CT20 light exposure. Per2 and Per3 expression only decreased during the CT20 exposure, whereas Dbp expression was increased during the CT14 exposure. In addition to these changes in clock gene expression, the expression of AANAT, an output gene involved in melatonin production, showed a decrease after light exposure at both time points, with a pronounced effect at CT20, i.e., a time point at which endogenous AANAT expression is high (**Figure 3**). The correlation analysis for clock gene *versus* AANAT (output) revealed: 1) a significant (positive) correlation for Per1 and AANAT at CT15 (R_2_ = 0.51, p = 0.036) and an almost significant correlation at CT21 (R_2_ = 0.60, p = 0.051), 2) a positive correlation between Per2 and AANAT at CT15 (R_2_ = 0.51, p = 0.035), but not at CT21 (R_2_ = 0.45, p = 0.16), 3) and a positive correlation between Per3 and AANAT at CT21 (R_2_ = 0.62, p = 0.037) but not at CT15 (R_2_ = 0.31, p = 0.22). Finally, we found a negative correlation between Dbp and AANAT gene expression at CT15 (R_2_ = −0.59, p = 0.02), but not at CT21. Interestingly, whereas the AANAT response was identical at both CT points, the clock genes that correlated with AANAT gene expression did not do so consistently.

Although the adrenal, like the pineal, is innervated by the sympathetic branch of the autonomic nervous system, the clock gene expression in this organ displayed a completely different pattern after light exposure. In the adrenal, statistical analysis indicated a significant increase of Cry2 expression at CT20 only. On the other hand, the expression of MC2R, i.e. the ACTH receptor, increased significantly with light at CT14 but not at CT20 (**Figure 3**), matching the absence of corticosterone changes at CT20 [9], [10]. The different effects of light exposure observed on these two sympathetic innervated tissues probably reflects the tissue- specific sympathetic outflow as previously described by [38]. Significant correlations between clock gene expression and that of the MC2R gene were observed at CT15, for Per3 (R_2_ = 0.57, p = 0.016), Cry1 (R_2_ = 0.75, p<0.001) and Dbp (R_2_ = 0.777, p<0.001). Interestingly, however, in contrast to MC2R, none of the changes in clock gene expression **were** significant.

### Response of liver, heart and muscle to light exposure

In the liver, light exposure caused a significant increase of Per1 and Per2 expression at CT14. The significant Group * Treatment interaction (**Table 1**) indicates that the Per1 and Per2 responses to light differ according to the time of the night, reinforcing the idea that the light pulse induces a time-dependent effect on these two clock genes **(** **Figure 1** **&** **2** **)**. No significant changes of other liver clock genes were observed after light exposure applied at CT14 or CT20 (**Figure 1** **&** **2** **,** **Table 1**).

Interestingly, light exposure also induced a change in the expression level of several gluco-regulatory enzymes (**Figure 3** **,** **Table 2**). PEPCK expression was increased both at CT14 and CT20, whereas GLUT2 expression was increased at CT14 and glucokinase (GCK) expression decreased at CT20. Analysis revealed some significant correlations between clock gene expression and that of the liver-glucose enzymes. The expression of Per2 correlated significantly with that of GCK at CT 20 (R_2_ = 0.84, p<0.001), but not CT 15. GLuT2 expression correlated significantly with Per1/2 at CT15 (R_2_ = 0.86, p<0.0001 and R_2_ = 0.91, p<0.001), but not at CT20, indicating a common pathway by which light can affect Per1/2 and GluT2 gene expression. However, despite the correlation observed for Cry1 and GLUT2 (R_2_ = −0.73, p<0.001) and for Dbp and GLUT2 (R_2_ = 0.79, p<0.001) only at CT15 do these 2 clock genes not respond to light as GLUT2. Significant correlations between clock gene expression and that of the PY gene were observed with Per1 (R_2_ = 0.64, p = 0.005) at CT15 but not CT20, with Per2 (R_2_ = 0.76, p<0.001 and R_2_ = 0.63, p = 0.027, respectively for CT 15 and 20), with Cry 1 at CT20 (R_2_ = 0.73, p = 0.006) and with Dbp at CT15 (R_2_ = 0.55, p = 0.021). Despite these significant correlations, the effect of light on PY expression was not significant. Finally, for PEPCK, despite the significant correlation at CT15 with a number of clock genes (R_2_ = 0.85, p<0,0001 and R_2_ = 0.91, p<0,0001, R2 = 0.61, p = 0.009 for respectively Per1/2 and Dbp), the light-induced increase in PEPCK transcripts at CT20 was not accompanied by significant changes in Per1/Per2 or Dbp at CT20.

In the heart, several clock genes responded to the light exposure. Light applied at CT14 triggered an upregulation of Per3 and Dbp, whereas light applied at CT20 induced a decrease in Per1, Per2 and Cry2 mRNA levels.

In the muscle, changes in clock gene expression were observed only when light was applied at CT20, when both Cry1 and Cry2 were upregulated (**Figure 1** **&** **2**).

In summary, light exposure affects the expression of clock genes in a differential manner, depending on the organ and the circadian time. Moreover, output genes in all tissues studied also showed time-dependent effects of light exposure. Surprisingly, correlation analysis of the light-induced clock gene and functional gene responses revealed a common effect of light exposure at both CT-times only for GluT2 and the Per1/2 genes.

### Role of the autonomic nervous system in the effect of light on liver metabolism

To investigate the role of the autonomic nervous system in signalling light information to the liver, we analyzed gene expression in the liver, pineal, heart and adrenal of groups of complete hepatic denervation (CD) and sham-operated animals with and without a 1 h light exposure at CT14. Clock gene expression in muscle tissue did not exhibit a clear effect of light applied at CT14 in Experiment-1, therefore this organ was not further analyzed in the CD or sham-operated animals.

### Effect of light on gene expression in CD animals

In our earlier study we reported an average noradrenaline (NA) concentration in the liver of 22.2±1.7 ng/g of liver tissue [28]. In the present study, the average NA concentration in four control animals was 25.2±3.6 ng/g of liver. In ten out of eleven CD animals the NA content was <2.1 ng/g (mean 1.06±0.39 ng/g). The remaining animal, which was discarded from the final analysis, had a NA content of 27.3 ng/g of liver. Six out of 10 CD animals were subjected to CT14 light exposure, whereas the 4 remaining ones were incorporated in the non-light exposure group. A complete denervation of the autonomic input to the liver had no overall effects on the basal expression level of clock genes and output genes. Only in 4 out of 31 combinations did ANOVA indicate a significant effect of *Treatment* (i.e. denervation): pineal Dbp, liver PEPCK, Per2 in the heart and MC2R in the adrenal (**Table 1** **&** **2**). However, none of these 4 remained significant in the post-hoc analysis (**Figures 1** **,** **2** **&** **3**). Therefore, liver denervation *per se* does not affect the basal expression of the different genes studied, confirming our previous study [28]. However, light-induced changes in the liver mRNA levels of clock genes and output genes were clearly affected by the removal of the ANS input (**Table 1** **&** **2** and **Figures 1** **,** **2** **&** **3**).

Hepatic denervation not only prevented the effect of light on the upregulation of Per1 and Per2, it also abolished the light-induced changes in PEPCK and GLUT2 mRNA expression. These data suggest that the ANS plays an important role in transmitting light information to different peripheral tissues.

In the pineal, the CD did not impair the decrease of Per1 expression or the upregulation of Dbp expression after a light exposure at CT14. Interestingly, the effect of light on Dbp upregulation appeared to be more pronounced in CD animals. In a similar manner, the inhibitory effect of light on AANAT expression appeared to be stronger in CD animals as compared to intact animals (**Figures 1** **,** **2** **&** **3** and **Table 1** **&** **2**).

In the adrenal of CD animals the light-induced upregulation of the MC2 receptor was absent.

### Effect of light on hormonal changes in CD animal

Since a complete denervation of the liver completely abolished the effect of nocturnal light on gene expression levels in the liver, but also on the MC2R changes in the adrenal, it was essential to measure the changes in plasma corticosterone and glucose concentrations in CD animals.

**Figure 4:**
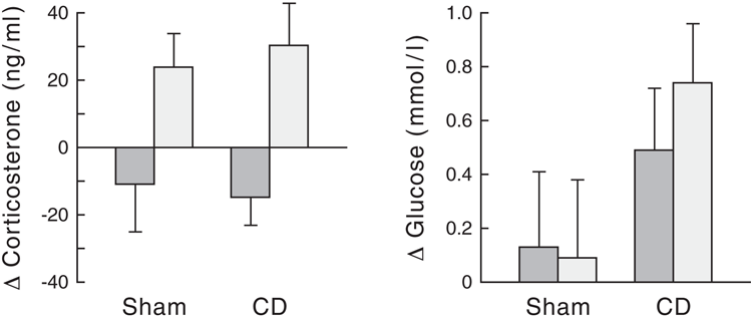
Effect of light at CT14 on hormonal levels in intact *vs* denervated animals. Changes in the plasma glucose/corticosterone levels after one hour of light exposure at CT14 in sham-operated (n = 8) and CD (n = 10) animals. One-way ANOVA with repeated measures (i.e., *Treatment* (2 levels: light *versus* no-light)) and, as between-animal factor, *Group* (2 levels: sham-operated *versus* completely denervated CD) were used to assess the changes in plasma glucose/corticosterone levels after one hour of light exposure. Dark bars represent the animals in the non light-exposed condition and white bars represent the same animals after light exposure. Each value is the mean±S.E.M of the delta value (T_0_–T_60 min_).

The data of 10 out of the 14 CD animals (i.e., one had a blocked catheter and 3 were discarded according to the corticosterone criterion, see statistical analysis paragraph) and 8 out of the 10 sham-operated animals (i.e., one animal had a blocked catheter and one was discarded according to the corticosterone criterion) were subjected to one-way ANOVA with repeated measurement. The analysis of the corticosterone data revealed a clear effect of light exposure (*Treatment*, p<0.001; **Figure 4**). The absence of a *Group* (p = 0.910) and Group*Treatment interaction effect (p = 0.670) indicates that the complete denervation of the liver did not affect the corticosterone response to light exposure. In contrast to corticosterone, no significant changes in plasma glucose concentrations were observed after one hour of light exposure (*Treatment*, p = 0.6; Treatment*Group, p = 0.456; *Group*, p = 0.114).

These results led to our conclusion that, despite the loss of an upregulation of MC2R, the light-induced corticosterone response is maintained in CD animals. Consequently, the lack of changes in PEPCK and GLUT2 mRNA levels after light exposure is due to the removal of the hepatic ANS input, not to the loss of the corticosterone response.

## Discussion

The present study demonstrates immediate effects of nocturnal light exposure on the peripheral tissues by means of rapid changes in the expression level of several (clock) genes in different organs. The light-induced changes in the expression patterns of (clock) genes are organ and time-of-day specific. In addition, we demonstrated for the first time the role of the ANS in the light-effect on peripheral organs, not only for the pineal and adrenal but also for the liver. Indeed, the changes in liver gene expression (Per1, Per2, GluT2 and PEPCK) observed after nocturnal light exposure are dependent on an intact input from the ANS and do not depend on changes in the secretion of corticosterone or melatonin. Moreover, not only has light a differential effect on clock gene expression in different organs, the changes in clock gene expression, too, do not correlate in a consistent manner with the changes in the expression of the output genes studied. Taking these observations together, a ‘decisive’ role of peripheral clock genes for the response to nocturnal light of the functional/output-genes within an organ is not immediately apparent. Furthermore, and more importantly, the autonomic innervation of the liver is essential for the transmission of the light information, most likely via the SCN, in order to adjust the liver metabolism immediately according to the light changes in the environment.

### Role of the ANS in nocturnal effect of light on the adrenal

The neuronal pathways connecting the SCN to the adrenal are involved in the increase of the corticosterone secretion after prolonged exposure to light at CT14 [10]. It was demonstrated that adrenal responses to light exposure in the early hours of the night occurred without changes in the release of adrenocorticotropic hormone (ACTH) or the adrenal hormones adrenaline and noradrenaline [9], [10]. ACTH acts on adrenocortical cells and promotes steroidogenesis by specific binding to the ACTH (MC2R) receptor [39]. In the present study we demonstrated that MC2R mRNA levels were significantly increased after light exposure at CT14, but not at CT20. This observation fits in well with the fact that plasma corticosterone levels were increased at CT14 (but not at CT20) without any noticeable changes in ACTH release [9], [10].

However, additional experiments should be performed to demonstrate whether the photic regulation of the MC2R mRNA also reflects the ACTH receptor density of the adrenal. Surprisingly, we observed a secretion of corticosterone after light exposure at CT14 in CD animals, despite the absence of an increased of MC2R expression. These data suggest that light is able to influence corticosterone synthesis independently of a change in the MC2R, maybe via a direct effect on the rhythmic expression of enzymes involved in the steroid synthesis [40]. All these observations indicate a more complex control of the SCN on the adrenal by the autonomic innervation; therefore further experiments will have to be conducted to elucidate the exact mechanisms involved in the effects of light on corticosterone secretion.

### Role of the ANS in the nocturnal effect of light on the liver

In the present study, we observed changes in peripheral clock gene/output gene expression in some organs that appeared to be correlated with the light-induced changes in the plasma melatonin [11], [41] and/or corticosterone levels [10], [13]. For instance, the upregulation of Per1, Per2, and GLUT2 expression in the liver at CT14 can be correlated with the increase in corticosterone secretion, whereas the decrease in GCK mRNA expression might be ascribed to the shutdown of melatonin secretion after light exposure at CT20. These results indicate that organs may respond to more than one hormonal cue, depending on the time-of-day at which the light is applied. On the other hand, in our study we also observed responses that could not be correlated with the changes in plasma melatonin or corticosterone. For example, the similar response of PEPCK mRNA after light exposure at CT14 and CT20 cannot be explained by corticosterone changes, but indicates that light information is transmitted by another pathway. Therefore, we propose that the ANS participates in immediate light-induced changes in the liver metabolism, as already demonstrated for the pineal and the adrenal [10], [42]. In fact, we clearly showed that in the absence of the autonomic input to the liver, the light-effect at CT14 on Per1/Per2, GLUT2 and PEPCK expression was completely abolished, indicating that these effects of light on the liver are mediated by the ANS. In addition, since in CD animals the effect of light exposure on corticosterone secretion is not altered, this observation confirms the role of the ANS in light-induced liver gene responses. It is not clear, however, whether the presently observed changes in the expression patterns of the output genes also result in functional changes of liver activity. Many studies, including our own, have reported a correlation between the daily patterns of liver gene expression and liver enzyme activity [43]–[46]. In addition, recently a clear correlation has been demonstrated between Pepck gene expression and hepatic glucose output [47], [48]. Together, these observations make it very likely that the presently observed changes in the gene expression of liver enzyme after light exposure will also result in a change in liver function, i.e. an increase in hepatic glucose production. On the other hand, the absence of changes in plasma glucose levels in the light-exposed group does not seem to support the above conclusion. However, plasma glucose concentrations do not accurately reflect changes in hepatic glucose metabolism, as this parameter is a resultant of changes in both glucose production and glucose uptake. Therefore, isotope dilution studies will be needed to evaluate the effects of nocturnal light exposure on hepatic glucose production and peripheral glucose uptake in more detail [49].

### Autonomic control of liver metabolism: new perspective

The immediate effects of light on pineal melatonin release and the neuro-anatomical connections involved have been extensively studied by microdialysis, brain lesions and neuronal tracing techniques [24], [42], [50]–[52]. The outcome of these studies revealed that 1) photic activation of the SCN neurons triggers a release of GABA onto the paraventricular nucleus of the hypothalamus (PVN) and thereby inhibits the activity of the pre-autonomic neurons and consequently stops melatonin production/secretion in the pineal gland [50], [51] and 2) a balanced stimulatory/inhibitory input from SCN to the pre-autonomic neurons located in the PVN generates the daily melatonin rhythm [42], [53].

Interestingly, the control of the daily glucose metabolism in the liver also indicates an important role for the GABAergic/Glutamatergic SCN inputs to the hypothalamic pre-autonomic neurons that are connected to the liver [54], [55]. In the present study, we clearly demonstrated a direct effect of nocturnal light on hepatic genes involved in glucose metabolism via the ANS. Therefore, it would be interesting to investigate further how light-activation of SCN neurons affects the activity of the two branches of the ANS that adjust glucose metabolism in the liver.

The present study clearly shows that light affects liver metabolism also through autonomic input and not solely via light-induced corticosterone secretion. Previously we have shown that the SCN controls plasma glucose concentrations via the sympathetic innervation to the liver [54]. Our present observation, that 1 hour of nocturnal light exposure can also affect the expression level of certain gluco-regulatory enzymes in the liver, illustrates the sensitivity of peripheral organs, e.g. the liver, to autonomic input and hence to the output of the SCN.

Furthermore, our study suggests that the SCN transmits light information to the peripheral oscillators in a differential manner according to the organs, i.e., endocrine and/or neuronal signals. Indeed, such a differential control of the SCN over the peripheral clocks has also recently been reported by using the parabiosis technique to discriminate between the neural and humoral SCN output [56]. Finally, as recently demonstrated for the adrenal and the respiratory tract [10], [20], our results show that also in case of the liver the autonomic nervous system is essential for conveying light information in order to synchronize the hepatic function according to the time of the day.

The lack of light-effect on the MC2-R expression in CD animals and also the more pronounced light-inhibition on AANAT expression in CD animals suggest that interference with the autonomic innervation of the liver may result in a disturbed autonomic output to other organs as well. Although we are not aware of any other evidence indicating similar effects of liver denervation on the adrenal or pineal gland, there are indications in the literature that affecting one organ may affect the function of other organs by mechanisms yet unknown. For example, pinealectomy induces changes in autonomic activity towards the heart even though melatonin treatment [57] and liver dysfunction influence peripheral organs, such as adipose tissue, via the vagal afferent nerve [58].

### Peripheral clock genes: the same molecular mechanism as within the SCN?

The molecular mechanism of SCN clock function, composed of several rhythmically expressed genes, consists of interacting positive and negative transcription/translation feedback loops [59]. Besides the SCN, clock genes are also rhythmically expressed in peripheral tissues, suggesting a possible universal role of clock genes to mediate temporal control over physiological diurnal events in peripheral organs.

In this respect, in the present study we observed that light affects clock gene expression in a differential manner, not only depending on the time-of-day but also, and more interestingly, depending on the organ. For instance, nocturnal light at CT15 triggers changes of Per's gene expressions in the liver, pineal and heart but not in the adrenal and the muscle. Some clock genes in the adrenal and pineal did not show a similar response to light, which is surprising, as both these organs are innervated by the sympathetic branch of the autonomic nervous system. This discrepancy in the effect of light on the different organs might be explained by the capacity of the peripheral oscillator to respond to various cues (e.g. chemicals [60], [61], hormones [62] and temperature [63]). Therefore, we propose that the resetting mechanism of the peripheral oscillators may differ from the SCN as well as from one organ to another. Indeed, the fact that Ishida and co-workers [10], using a similar light stimulus in mice, found a strong increase in Per1 in the adrenal and not in the liver, while in the present study we observed no changes in the Per mRNAs but did find an increase in Cry2, both in liver and adrenal, only emphasizes the variability of the responses of clock genes in the organs and suggests it might be species-dependent.

In the present study, we aimed to reveal a possible role of the clock genes in the immediate effect of light on the functionality of the organ. We found no clear correlation between the clock/functional genes, suggesting that some peripheral functions may be controlled by the SCN without the direct involvement of peripheral clock genes (at least in as far as can be seen from the mRNA level). Indeed, although some clock genes and AANAT in the pineal may share the same regulatory pathway, i.e. the multisynaptic pathway between SCN and pineal [16], [64], no causal relation has been shown between them, either in the present study or in a previous one [65]. ). In line with this idea, a recent study demonstrated that peripheral clock gene oscillations and their phase relation between organs were restored by SCN grafts despite “aberrant” locomotor activity (i.e., compared to intact animal) and disturbed endocrine rhythm [66], [67]. Interestingly, only a limited number of studies provided a real insight into the clock gene function, e.g., cell division [68], [69], chromatin remodeling [70] and haem metabolism [71]. These functions are mainly taking place at the cell level and so far no clear function has been demonstrated at the level of the entire organ. In summary, the mechanism that uses light as a means to reset appears to differ from one organ to another, indicating that the molecular clock mechanism in peripheral tissues needs to be further studied in order to reveal their role in the daily physiology.

In conclusion, the present results suggest that light-induced changes in SCN activity and output not only result in immediate changes in the pineal and adrenal, but also affect the physiology of the heart and liver. Moreover, the SCN may use multiple pathways to transmit these light-induced changes to the rest of the body, but direct signaling via the ANS plays an essential role, enabling the SCN to target its message to specific organs.

## Materials and Methods

### Animals

Male Wistar rats (Harlan Nederland, Horst, The Netherlands) were housed at room temperature of 21±1°C with a 12-h light, 12-h dark (L/D) cycle (lights on at 07:00 AM; lights on being defined as Zeitgeber Time 0). Room lights had an intensity of ≈200 lux, while under dark conditions the dim red light was <1 lux. Animals were housed four to six per cage and transferred to individual cages (25×25×35 cm) after surgery or 1 week prior to the light exposure experiment. One day before the experiment, all groups of rats (i.e., no light and light-exposed groups) were placed under D/D regimen. Light applied at CT14 and CT20 had an intensity of ≈200 lux. Food and water were available *ad libitum*.

Ethics Statement: All of the following experiments were conducted under the approval of the Animal Care Committee of the Royal Netherlands Academy of Arts and Sciences.

### Surgical procedure

Surgery was performed in rats of 300–350 g. During all surgical procedures animals were anesthetized using a mixture of Hypnorm (Duphar, The Netherlands; 0.05 ml/100 g body weight, i.m.) and Dormicum (Roche, The Netherlands; 0.04 ml/100 g body weight (BW), s.c.). The wound was closed with traumatic sutures (5-0 Perma-Hand Said Ethicon), and postoperative care was provided with a subcutaneous injection of 0.01 ml/100 g BW of painkiller (Temgesic; Schering-Plough (Utrecht importer)). The animals were allowed a recovery period of at least 10 days so that they could regain their pre-operative body weight and circadian rhythms.

#### Complete denervation of liver

By using microsurgical techniques, a complete hepatic denervation CD (i.e. sympathectomy and parasympathectomy) was performed, after a laparotomy had been performed in the midline.

Hepatic sympathectomy was performed as previously described [28], [54]. Briefly: the ligaments around the liver lobes were severed to free the bile duct and the portal vein complex. The nerve bundles were identified at the level of the hepatic artery proper and a 0.5 cm was removed using microsurgical instruments.

Hepatic parasympathectomy was also performed as previously described [46], i.e., the hepatic branch was revealed where it separated from the left vagal trunk, and subsequently the fascia containing the branch was stretched and then sectioned with a pair of scissors. The disruption of the hepatic vagus branch close to the oesophagus (before the entrance to the liver) ensures a complete vagal hepatic denervation [46], [72], [73].

#### Jugular vein catheter

A jugular vein catheter was implanted to investigate the hormonal response of the adrenal cortex to light exposure (i.e., corticosterone secretion) at different time points. During the surgical procedure, an intra-atrial silicone cannula was implanted through the jugular vein according to the method of Steffens [74] to allow stress-free remote blood sampling.

### Experimental set up

Different groups of animals were treated simultaneously as follows: at CT14, two groups of animals were exposed to 60-min light (intact and completely denervated (CD)). Control groups (i.e., liver-intact and CD) did not receive light exposure. All groups were sacrificed at CT15. In parallel, additional groups were provided with a jugular vein catheter to monitor the hormonal changes. The day of the control experiment (no light), blood samples were taken at CT14 (time = 0 min) and CT15 (time = 60 min), in sham-operated and CD animals. One week later, the blood sampling experiment was repeated with the same groups of animals, but this time with the lights on from CT14 until CT15. At CT20, one group of intact rats was exposed to 60-min of light, whereas a group of non-exposed animals served as control. Both groups were sacrificed at CT21.

### End of the experiment

Animals were sacrificed immediately at the end of the light exposure (at CT15 or CT21). Pineal, adrenal, liver, heart and skeletal muscle (triceps brachii) were removed and frozen directly into liquid nitrogen and transferred to −80°C until analysis.

### High Performance Liquid Chromatography-electrochemical measurements

We checked the completeness of the sympathectomy by measuring the NA content in the liver. Pieces of liver were processed using High Performance Liquid Chromatography (HPLC) with electrochemical detection for NA content according to our published method [28]. Sympathetic denervation was considered to be complete when the NA content dropped below 10% of that of intact-liver animals [75].

### RNA extraction and cDNA synthesis

Total RNA was extracted and purified by RNeasy mini kit (Qiagen, Courtaboeuf, France) including a DNase step, according to the manufacturer's recommendations. The quality of RNA was examined by Agilent 2100 Bioanalyzer equipped with Nano chips (Agilent Technologies, Palo Alto, CA, USA) and concentrations were determined by Nanodrop spectrophotometer (NanoDrop Technologies, USA). 2 µg (liver, adrenal, muscle and heart) or 0.5 µg (pineal) of total RNA was reverse-transcribed in a final reaction volume of 20 µl. First, total RNA and random hexamer (125 ng/µg of RNA, Hexanucleotide Mix, Roche, Life Technology Nederland) were incubated for 10 min at 70°C. Then, 5× first strand buffer, 0.1 M DTT and dNTPs (10 mM) were added and the samples were preincubated for 2 min at 42°C. Finally, 1 µl of Superscript RNase H^−^ Reverse transcriptase (SSIIRT, In Vitrogen, Life Technology Nederland) was added and the reaction was incubated for one hour at 42°C. For the control sample (no RT), the 1 µl of enzyme was replaced by 1 µl of water.

### Real-time PCR (RT-PCR)

We analyzed the gene expression of clock/output genes by RT-PCR (Applied Biosystems, Model ABI7300 Prism Sequence Detection System). 1 µl of each cDNA was incubated in a final volume of 20 µl RT-PCR reaction containing 10 µl of 2× SYBR-Green master mix, 1.5 µl of each primer at 3 pmol.µl^−1^ and 6 µl of RNase-free water. Quantitative PCR was performed under the following general PCR conditions: 2 min at 50°C, 10 min at 95°C, followed by 40 cycles of 15 s at 95°C; 1 min at 60°C. The data were acquired and processed automatically by Sequence Detection Software (Applied Biosystems Inc). Gene-specific primers were designed using Primer Express Software (Applied Biosystems, Foster City, CA, USA). The primer data are given in **Table 3**. The reference genes mentioned (H1, HPRT, GAPDH, Ubi, Tbp, EFalpha) were tested for each organ on 12 to 15 samples. Reference genes were selected based on their constant expression under the various experimental conditions (i.e., light and/or denervation). From the reference genes that fulfilled the RT-PCR criteria for each organ we selected the 2 most stable reference genes with the GeNorm v3.3 program (http://allserv.ugent.be/~jvdesomp/genorm/), for more details, see [28]. In brief, Histone1 (*H1*) and hypoxanthine-guanine phosphoribosyl transferase (*HPRT*) were chosen as reference genes for pineal; glyceraldehyde-3-phosphate dehydrogenase (*GAPDH*) and ubiquitin (*Ubi*) for adrenal, *HPRT* and *Ubi* for heart, TATA box binding protein (*Tbp*) and *Ubi* for liver, and *H1* and elongation factor alpha (*EFa*) for muscle. Primer specificity was confirmed by dissociation (melting) curve analysis and 5% polyacrylamide gel electrophoresis with 25 bp ladder.

**Table 3:** Primer sequences for RT-PCR.

Gene	Gene bank	Sequence
Histone 1	X70685	5′ GAACGCCGACTCCCAGATC3′
		5′ CCCCTTTGGTTTGCTTGAGA3′
HPRT	AF009656	5′ATGGGAGGCCATCACATTGT3′
		5′ATGTAATCCAGCAGGTCAGCAA3′
GAPDH	NM_017008	5′TGCCAAGTATGATGACATCAAGAAG3′
		5′AGCCCAGGATGCCCTTTAGT3′
Ubi	NM_025356	5′CTCCAACAGGACCTGCTGAAC3′
		5′CTGAAGAGAATCCACAAGGAATTGA3′
Tbp	XM 217785	5′ TTCGTGCCAGAAATGCTGAA3′
		5′TGCACACCATTTTCCCAGAAC3′
EF1a	NM_012660	5′AGATGGACTCCACGGAACCA3′
		5′GTAGGCGCTGACCTCCTTGAC3′
Per1	AB092976	5′TCTGGTTCGGGATCCCACGAA 3′
		5′GAAGAGTCGATGCTGCCAAAG3′
Per2	NM_031678	5′CACCCTGAAAAGAAAGTGCGA3′
		5′CCACGCCAAGGAGCTCAAGT3′
Per3	NM_023978	5′ATAGAACGGACGCCAGAGTGT3′
		5′CGCTCCATGCTGTGAAGTTT3′
Cry1	NM_007771	5′AAGTCATCGTGCGCATTTCA3′
		5′TCATCATGGTCGTCGGACAGA3′
Cry2	NM_133405	5′TGGATAAGCACTTGGAACGGAA3′
		5′TGTACAAGTCCCACAGGCGGTA3′
Dbp	NM_012543	5′CCTTTGAACCTGATCCGGCT3′
		5′TGCCTTCTTCATGATTGGCTG3′
AANAT	NM_012818	5′TGAGCGCGAAGCCTTTATCTC3′
		5′TGATGAAGGCCACAAGACACC3′
G6pc	NM_013098	5′CCCATCTGGTTCCACATTCAA3′
		5′GGCGCTGTCCAAAAAGAATC3′
GLUT2	NM_012879	5′GAAGGATCAAAGCCATGTTGG3′
		5′CCTGATACGCTTCTTCCAGCA3′
GCK	NM_012565	5′TCCTCCTCAATTGGACCAAGG3′
		5′TGCCACCACATCCATCTCAA3′
PY	NM_012624	5′GAGAGTTTTGCAACCTCCCCA3′
		5′CCTTCACAATTTCCACCTCCG3′
PEPCK	NM_198780	5′TGCCCTCTCCCCTTAAAAAAG3′
		5′CGCTTCCGAAGGAGATGATCT3′
MC2R	AF547168	5′CCTTCTGCCCAAATAACCCTT3′
		5′CATGCCATTGACCTGGAAGA3′

#### Detection of the clock/output gene expression by the RT-PCR

The absolute level of mRNA for the clock/functional genes in each sample was quantified. The relative expression of these genes was obtained by dividing the absolute amount of the target gene by the average of the two reference genes values.

### Plasma measurements

Plasma corticosterone concentrations were determined in duplicate with a radioimmunoassay kit (ICN Biomedicals, Costa Mesa, CA). From the samples, 10 µl was taken and diluted in 4 ml of assay buffer. The lower limit of the assay was 1 ng/ml and the coefficient of variation of the immunoassay was less than 4%. Plasma glucose concentrations were determined using a glucose/glucose oxidase-Period method (Boehringer Mannheim, Mannheim, Germany) as previously described [76].

### Statistical analysis

RT-PCR data were analyzed by a mixed one-way ANOVA. If significant effects were detected, the ANOVA was followed by a post-hoc LSD test (for more details see legends of Tables 1 & 2). Plasma data are presented as a mean of Δ values (T_60 min_−T_0 min_)±SEM. One-way ANOVA with repeated measures was used to assess the changes in plasma glucose/corticosterone concentrations after one hour of light exposure (see legend Fig. 3 for more details). Animals with plasma corticosterone concentrations >150 ng/ml (i.e. the normal value of the daily peak) at T = 0 min were discarded for further data analysis. Pearson correlation was used to test for associations between clock gene and output gene expression. Differences were considered significant when *p* values were lower than 0.05.
